# The Yin and Yang of Pneumolysin During Pneumococcal Infection

**DOI:** 10.3389/fimmu.2022.878244

**Published:** 2022-04-22

**Authors:** Joana M. Pereira, Shuying Xu, John M. Leong, Sandra Sousa

**Affiliations:** ^1^ i3S-Instituto de Investigação e Inovação em Saúde, Universidade do Porto, Porto, Portugal; ^2^ Instituto de Biologia Molecular e Celular, Universidade do Porto, Porto, Portugal; ^3^ Molecular and Cellular (MC) Biology PhD Program, ICBAS - Instituto de Ciência Biomédicas Abel Salazar, University of Porto, Porto, Portugal; ^4^ Department of Molecular Biology and Microbiology, Tufts University School of Medicine, Boston, MA, United States; ^5^ Graduate Program in Immunology, Tufts Graduate School of Biomedical Sciences, Boston, MA, United States

**Keywords:** *Streptococcus pneumoniae*, pore-forming toxin, cholesterol-dependent cytolysin, plasma membrane, pneumonia, pro- and anti-inflammatory immune responses, pneumolysin

## Abstract

Pneumolysin (PLY) is a pore-forming toxin produced by the human pathobiont *Streptococcus pneumoniae*, the major cause of pneumonia worldwide. PLY, a key pneumococcal virulence factor, can form transmembrane pores in host cells, disrupting plasma membrane integrity and deregulating cellular homeostasis. At lytic concentrations, PLY causes cell death. At sub-lytic concentrations, PLY triggers host cell survival pathways that cooperate to reseal the damaged plasma membrane and restore cell homeostasis. While PLY is generally considered a pivotal factor promoting *S. pneumoniae* colonization and survival, it is also a powerful trigger of the innate and adaptive host immune response against bacterial infection. The dichotomy of PLY as both a key bacterial virulence factor and a trigger for host immune modulation allows the toxin to display both “Yin” and “Yang” properties during infection, promoting disease by membrane perforation and activating inflammatory pathways, while also mitigating damage by triggering host cell repair and initiating anti-inflammatory responses. Due to its cytolytic activity and diverse immunomodulatory properties, PLY is integral to every stage of *S. pneumoniae* pathogenesis and may tip the balance towards either the pathogen or the host depending on the context of infection.

## 1 Introduction

*Streptococcus pneumoniae* (*Sp* or pneumococcus) is an extracellular Gram-positive bacterium that asymptomatically colonizes the upper respiratory tract of 5–10% healthy adults and 20–40% of children (1). *Sp* was considered the major cause of lower respiratory infections (LRI) with a global incidence of 2.7% worldwide, resulting in more than a million deaths per year and thus ranking among the deadliest bacteria (2). In the United States alone, pneumococcal pneumonia leads to over 150,000 annual hospitalizations. Other forms of *Sp* infectious complications such as otitis media, bacteremia, and meningitis are also significant healthcare burdens, leading to an average of 5 000 000, 4 000, and 2 000 cases/year respectively (source CDC: https://www.cdc.gov/pneumococcal/clinicians/clinical-features.html). Elderly and children under 5 are particularly susceptible to severe disease, with an invasive pulmonary disease (IPD) incidence of 7%. A major health concern and economic burden, *Sp* infection results in over $17 billion in direct medical costs annually in the US (3, 4).

*Sp* is naturally transformable and displays high genome plasticity, contributing to the rapid emergence of antibiotic resistance and evasion of vaccine-mediated immunity (1, 5). Effective *Sp* prevention and treatment is complicated by the appearance multidrug-resistant infections, non-vaccine serotypes and aging of the world’s population (4, 6). Approximately 30% of *Sp* cases involved isolates resistant to one or more antibiotics (https://www.cdc.gov/drugresistance/pdf/threats-report/strep-pneumoniae-508.pdf). Worldwide, pneumococci resistant to penicillin, erythromycin, and trimethoprim-sulfamethoxazole are on the rise (7). To a lower extent, resistance to tetracycline, chloramphenicol and fluoroquinolone have also been identified (8). While in the United States, multi-drug resistant pneumococci prevalence has been reduced since introduction of pneumococcal vaccines, the risk remains high in susceptible populations, especially individuals aged 65 and over (9). Vaccination has been the cornerstone of pneumococcal disease prevention. However, currently, the two classes of pneumococcal vaccines, the 23-valent pneumococcal polysaccharide vaccine (PPSV23) and the pneumococcal vaccines based on protein-conjugated polysaccharides (PCV13/PCV23), only protect against a subset of over 90 different pneumococcal capsular variants (capsular serotypes) (10). Through both the expansion of pre-existing non-vaccine pneumococcal serotypes and serotype ‘switching’, an exchange of capsular polysaccharide genes through transformation, infectious strains not covered by standard vaccination are on the rise (11). As the efficacy of traditional antibiotics and vaccines become compromised, understanding the mechanisms of action of pneumococcal virulence determinants is of critical importance for the development of new therapeutics.

**Figure 1:**
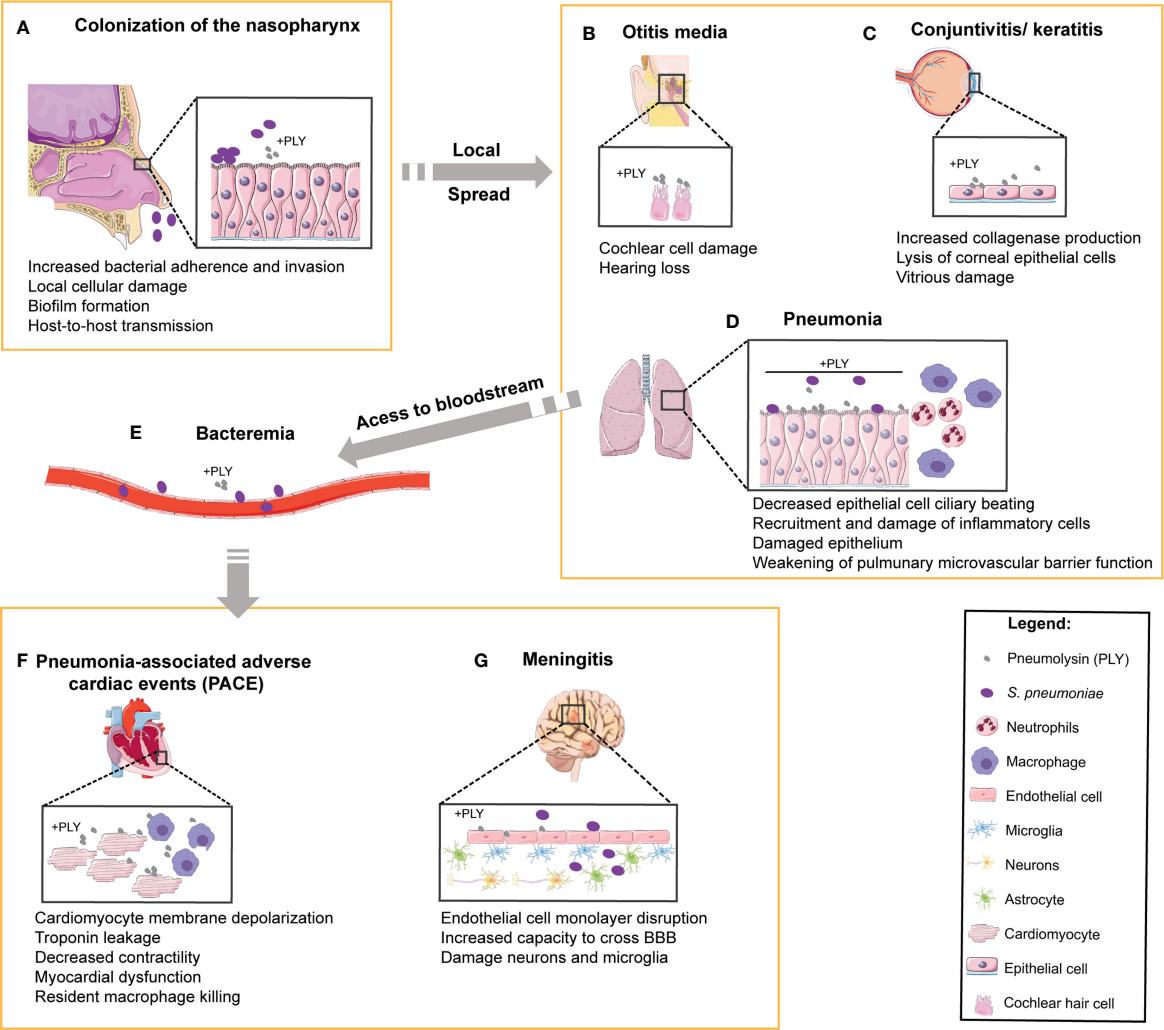
Role of PLY in human pneumococcal pathogenesis and in the targeting of multiple organs. In the multiple steps of pathogenesis during human infection by *S. pneumoniae*, the target organs and the cell types involved are indicated and the reported functions of PLY are listed below each step. **(A)** *S. pneumoniae* colonizes the human upper respiratory tract. Prolonged colonization of the nasopharynx may favor bacterial spread to neighboring tissues such as **(B)** the middle ear, causing otitis media and massive damage of cochlear hair cells, and **(C)** the eyes, leading to the development of keratitis and endophthalmitis. Upon aspiration, *S. pneumoniae* can reach **(D)** the lower respiratory tract and cause pneumonia. In the lungs, PLY causes the dysfunction of epithelial barrier, facilitating bacterial access to **(E)** the bloodstream, where PLY diminishes phagocytosis and disrupts endothelial/epithelial cells to promote tissue invasion. The bacteria are then able to infect **(F)** the heart, causing pneumonia-associated adverse cardiac events (PACE) by targeting the cardiomyocytes and resident macrophages, and **(G)** the brain, causing damage of both neurons and microglia.

*Sp* transmits predominantly *via* aerosol as the bacteria is harbored in the nasopharynx. Asymptomatic *Sp* carriage can lead to localized infection of tissues contiguous with the nasopharynx, causing sinusitis, otitis media, and pneumonia (1). The bacterium is also capable of subsequent systemic spread to the heart and brain upon access to the bloodstream, causing serious diseases such as cardiac dysfunction and meningitis (1) (**Figure 1**). The versatility of *Sp* is reflected by the diversity of interactions with its host depending on the site of infection and degree of disease. To colonize the host nasopharynx, *Sp* forms biofilms on the mucosa of the upper respiratory tract (12). This causes mucosal inflammation which promotes bacterial shedding in secretions leading to transmission (13). Long-term asymptomatic nasopharyngeal carriage supports inflammation-induced transmission and predisposes the host to develop disease (**Figure 1A**). *Sp* can then spread from the nasopharynx to neighboring tissues, such as the sinusoidal cavities to cause sinusitis, the middle ear to cause otitis media, or the eye to cause keratitis (**Figures 1B, C**), all of which can be recurrent infections especially in immunocompromised patients (14). *Sp* aspiration into the lower respiratory tract causes pneumonia, where infection damages alveolar epithelial and endothelial cells promoting tissue permeability and induces thrombotic events (**Figure 1D**). If the immune response mounted in the lungs is not sufficient to eliminate the bacteria, *Sp* may gain access to the bloodstream and disseminate to cause IPD (**Figures 1E** **–G**). During IPD, *Sp* may invade the spleen (15), the heart (16–18), and/or may cross the blood-brain barrier (19). In the heart, *Sp* forms biofilms and damages both cardiomyocytes and immune cells, leading to myocardial dysfunction and long-term pneumonia-associated adverse cardiac events (PACE; **Figure 1F**). *Sp*-induced remodeling of brain tissue during meningitis likely accounts for permanent neurological damage reported in about 50% of the survivors (**Figure 1G**).

Integral to all stages of *Sp* pathogenesis is the host-pathogen interaction mediated by pneumolysin (PLY), a key virulence factor produced by almost all clinical isolates and currently known *Sp* serotypes (20). PLY can promote asymptomatic carriage, colonization, transmission, immune evasion and dissemination, highlighting the multifaceted functions of this bacterial toxin. As a member of the cholesterol-dependent cytolysin (CDC) family, PLY is a pore-forming toxin (PFT) that at lytic concentrations disrupts host cell plasma membrane (PM) and promotes the uncontrolled influx and efflux of ions, small molecules, and proteins (21). PLY-induced pore formation interferes with a plethora of cellular signal transduction pathways. Depending on PLY concentration, which dictates the extent of PM damage and intracellular Ca^2+^ overload, host cells respond by activation of either cell death or cell survival and damage repair pathways. At lytic levels, PLY-induced pores can overwhelm cellular homeostatic mechanisms, triggering both cellular proinflammatory signaling pathways and irreversible cellular injury that results in the release of molecules heightening inflammation. The ensuing tissue damage is integral to pneumococcal disease (22). For example, in the lung, exacerbated inflammation promotes tissue invasion and systemic spread (21, 23). In contrast, at the sub-lytic concentrations likely present during the early stages of infection, PLY stimulates cell-autonomous repair mechanisms that overcome PM damage, restore cell integrity, and promote cell survival (24, 25), curtailing inflammation. In addition, depending on infection dose, site, disease stage, target cell, and the immune state of the host, PLY may display either “Yin” or “Yang” components, each with potentially opposing effects on *Sp* pathogenesis. The Yin of PLY promotes disease by promoting cell damage either directly or by excessive inflammation. The Yang of PLY mitigates the damage by triggering host repair mechanisms and modulating inflammation, allowing the carriage of *Sp* without symptoms. The remarkable ability of PLY to trigger both “Yin” and “Yang” responses depending on infection context is showcased in several *in vitro* and *in vivo* infection models (26, 27). Interestingly, heterogeneous expression of PLY within individual bacteria of an isogenic *Sp* population favors the appearance of subpopulations expressing different levels of PLY. Recent studies showed that genetically modified PLY expressing *Sp* strains producing different levels of PLY triggered different outcomes during infection. Specifically, high-PLY producing bacteria induced extensive autophagosome damage allowing efficient clearance of *Sp* from the host cells, yet, low-PLY producing bacteria facilitated evasion of host defense mechanism and promoted the cross of the blood-brain barrier (28, 29). Furthermore, rapid and high levels of PLY release drives hypervirulence in infection by serotype 1 pneumococci (30, 31), showcasing that the plasticity of PLY expression during *Sp* pathogenesis is fundamental and can influence the outcome of the infection.

Here we provide a comprehensive review of PLY functions and interactions with host cells by describing the mechanism of pore formation, its multiple targets and effects on host cells, and its antipodal interactions with the host immune system. We summarize the available data and recent discoveries that highlight the “Yin” and “Yang” perspectives of PLY activity contributing to *Sp* disease progression, unveiling the myriad and sometimes conflicting properties of this fascinating bacterial toxin.

## 2 Structural and Functional Insights of Pneumolysin

### 2.1 PLY Export

Secreted PLY is key for infection; however, the localization and export mechanism of PLY is still debated (32). PLY can be detected in the bacterial cytosol (32, 33), non-covalently attached to the cell wall (32), in culture supernatants (32), and in circulation during *Sp* infection (34). Given the lack of a canonical N-terminal signal peptide that promotes protein secretion, PLY was thought to be released solely by autolysis, an idea supported by experimental data showing reduced PLY release in the presence of non-bacteriolytic antibiotics (35). Nevertheless, autolysis may not be the only mechanism for PLY secretion (32, 36). In fact, although attenuated for virulence, *Sp* strains lacking autolysin release wild-type levels of PLY when cultured *in vitro* (37, 38). In addition, a functional SecA2 system is required for the non-covalent association of PLY to cell wall peptidoglycan (39), a structure that can hinder PLY delivery to the bacterial surface (40). Indeed, enzymatic digestion of *Sp* peptidoglycan promotes PLY release and is detrimental for virulence (40). Thus, PLY secretion is likely determined by several mechanisms that may cooperate to foster disease (31).

### 2.2 Mechanism of PLY-Mediated Membrane Permeabilization

**Figure 2:**
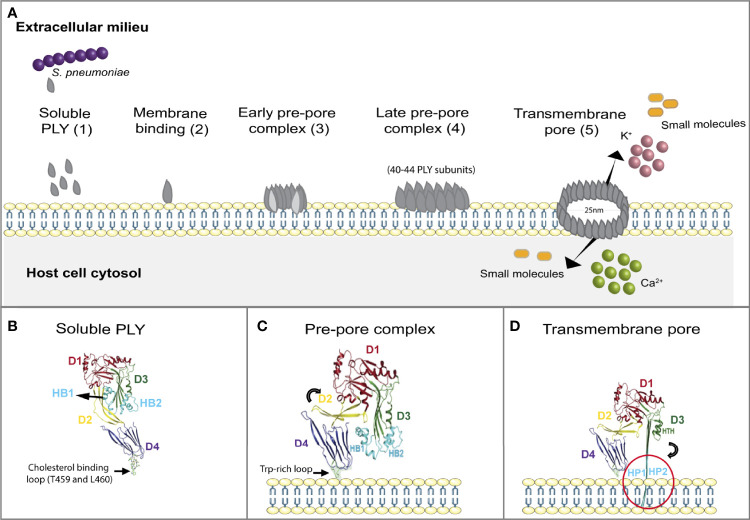
Mechanisms of PLY-mediated host plasma membrane permeabilization and conformational changes associated to pore formation. **(A)** PLY pore-formation is a multi-step process. PLY is released by *S. pneumoniae* as a water-soluble monomer (1) which specifically bind to cholesterol residues on the host cell plasma membrane (2). PLY monomers oligomerize by interacting with each other to form the early pre­ pore complex (3), which protrudes into the membrane surface establishing the late pre-pore (4). Finally, PLY inserts hairpins HP1 and HP2 across the membrane forming an open transmembrane channel, which allows the uncontrolled influx and efflux of ions and small molecules (5). **(B)** Soluble PLY. The 3D crystal structure of PLY monomer as it is released from *S. pneumoniae* is shown. The 4 major domains, from D1 to D4, as well as Helix Bundles (HB) 1 and 2 and cholesterol binding loop are indicated. The arrow indicates residues T459 and L460 in the D4 Trp-rich loop which are essential for cholesterol recognition. **(C)** PLY in pre-pore complex. Structure of PLY upon cholesterol binding via the conserved D4 Trp-rich loop. Interaction with cholesterol induces a 90° rotation of D2 (in yellow, indicated by the curved arrow) bringing D1and D3 towards the host plasma membrane. HB1 and HB2 are positioned just above the host membrane. This structural organization is maintained in the pre-pore stage. **(D)** PLY in the transmembrane pore. The 3D structure of PLY when inserted in the host cell plasma membrane is depicted. HB1 and HB2 refold into 85 Å β -hairpins HP1and HP2 (shown by the curved arrow), which insert (red circle) and cross the hydrophobic membrane to form an open transmembrane pore. Adapted from (42).

PLY shares structural and functional properties with other bacterial PFTs belonging to the CDC family, such as listeriolysin (LLO), perfringolysin (PFO), and streptolysin (SLO) (41). It binds to cholesterol residues at the PM of host cells, oligomerizes, and undergoes conformational changes to form stable pores with well-defined sizes (**Figure 2**).

To generate pores, soluble PLY interacts with the PM, multimerizes into two successive “prepore” complexes, and then inserts into the PM to generate a large pore (**Figure 2A**). Soluble PLY, in its monomeric state, has been shown by crystallographic methods to be an asymmetric molecule composed of 4 major domains (D1 to D4), including α-helices and β-sheets (**Figure 2B**). The N-terminal D1 domain, although not essential for cell binding, stabilizes overall protein structure and enables hemolytic activity (42, 43). The non-contiguous D1 and D3 are adjacent in the 3D crystal structure and linked to the C-terminal of D4 *via* D2 (44) (**Figure 2B**). Blocking monoclonal antibodies and the analysis of binding-defective point mutants indicate that D4 promotes membrane binding (21, 45–49). In particular, two residues (T459 and L460) in a conserved D4 undecapeptide that comprises a Trp-rich loop are essential for cholesterol recognition (21, 41, 50, 51) (**Figure 2B**, arrow). In addition, the recombinant PLY toxoid B “PdB” harboring a mutation in Trp 433 in D4 is unable to undergo the conformational changes required for membrane insertion and strains expressing this mutant show reduced virulence, further reinforcing the role of D4 in membrane binding and pore formation (13, 34).

PLY monomers multimerize, forming transient pre-pore asymmetric structures with variable diameters and composed of 40 to 44 monomers (**Figure 2A**) (44, 46, 52–56). D2 undergoes a 90° rotation, bringing D1 and D3 towards the PM, compacting the overall structure and establishing the late pre-pore (**Figures 2A, C**) (21, 53). PM insertion occurs when the D3 helix bundles (HB1 and 2) are refolded into 85A° β-hairpins (HP1 and HP2) that perforate the hydrophobic membrane and assemble into an approximately 260A° diameter transmembrane channel (**Figure 2D**) (52, 53). Mutations in D3 residues Y150 and T172 are found in a naturally occurring non-hemolytic variant of PLY (45, 57), pointing to their critical role in the conformational alterations required for transmembrane hairpin formation, PM insertion and subsequent pore-formation (45). Although it is unclear what advantage *Sp* derives from loss of PLY hemolytic activity, serotypes harboring naturally occurring non-hemolytic PLY are commonly associated with non-lethal respiratory tract infections (57) pointing to roles played by non-pore formation dependent mechanisms of PLY during *Sp* pathogenesis.

Additionally, it has been reported that PLY monomers may be trapped in arcs or slit-shaped oligomers (21) that have the ability to generate pores (58, 59), possibly with variable sizes, different permeabilities and distinct functional roles (55, 60). At low PLY concentrations, likely occurring at early stages of infection, complete prepore ring structures are expected to form less efficiently than arcs, and so pore formation may rely on the efficiency of the conversion of arc oligomers to pores. At high PLY concentrations, potentially reached later in infection, binding of soluble PLY to PM is efficiently followed by assembly of full rings, so cell lysis mainly depends on the PLY affinity for cholesterol (60). PM disruption following arc or full ring assembly possibly dictates different host responses according to the phase of infection and could influence the balance of Yin and Yang outcomes.

### 2.3 PLY as a Ligand for Host Molecules

In addition to binding cholesterol, PLY interacts with several other host molecules. Such interactions contribute to its functions as a both pro- and anti-inflammatory agent, further discussed in Section 4 below. First, through its D4 domain, PLY binds the mannose receptor C type 1 (MRC-1), expressed at the cell surface of multiple immune cell types in the airways (61). MRC-1 acts as an internalization receptor, allowing *Sp* to invade MRC-1-expressing dendritic cells (DCs) and alveolar macrophages. At low infectious doses when immune stimulation by bacterial PAMPs is limited, MRC-1-PLY interaction and the consequent *Sp* phagocytosis modulate inflammation (discussed in section 4.2.2) and promote intracellular bacterial survival in the lungs in a murine pneumococcal pneumonia model (61). Second, PLY also has the capacity to modulate the interaction of *Sp* with complement by functioning as a molecular decoy, binding the Fc portion of human IgG and triggering C1q recruitment and complement cascade activation (62, 63) (reviewed in section 4.2.4). This interaction is dependent on specific residues within short non-contiguous PLY sequences that are homologous to the human C-reactive protein (CRP), which also activates the complement (63). *In vivo*, diversion of complement proteins by PLY promotes both pulmonary and systemic infection, as PLY-deficient *Sp* become more virulent in complement-deficient mice (64). Lastly, PLY was suggested to bind host cell glycans such as sialylated fucosylated glycan divalent-LewisX (sLeX) (65), and computational docking studies suggested the D4 and the interface between D3 and D4 as putative binding sites for LeX and sLeX, respectively (46). Nevertheless, interactions detected *in vitro* utilized high sLeX : PLY ratios and thus appear to be of low affinity, and PLY:sLeX co-crystals have never been observed (46), indicating the need for further studies are needed to explore potentially biologically interactions interaction PLY and sLeX.

### 2.4 PLY-Mediated Pathogenesis and Targeted Cells

Due to its ability to bind to cholesterol commonly found in mammalian PMs, PLY is able to target and modulate the function of virtually all cell types and thus plays key roles in many modes of infection, such as asymptomatic carriage, local disease, or life-threatening systemic disease (66) (**Figure 1**).

Early in infection, PLY has vital roles in colonization of the nasopharynx and in host-to-host transmission (13, 67, 68) (**Figure 1A**). *In vitro*, PLY-deficient mutants are impaired in adherence (69) and show lower bacterial burden in the nasopharynx of intranasally infected mice (67, 68). *Sp* grown *in vitro* as a biofilm show increased *ply* transcription and produced high levels of PLY (70, 71), suggesting that *Sp* biofilms developed in the respiratory mucosa might also produce high levels of PLY. The PLY-induced mucosal inflammation promotes increased bacterial shedding in secretions and promotes transmission in an infant mouse model of *Sp* infection (13).

PLY is also a key molecule in the progression of *Sp* infection from the nasopharynx to neighboring tissues. A PLY-deficient mutant causes mild histopathological changes and lower middle ear bacterial loads in chinchilla OM model (72, 73). In guinea pig infection models, PLY directly damages cochlear hair cells (74, 75). In addition, PLY intracochlear perfusion induces a strong cytotoxic effect possibly related to pore formation at the PM of inner hair cells (76, 77) (**Figure 1B**). PLY also plays a role in pneumococcal ocular infections causing endophthalmitis and keratitis (78, 79). Intravitreally injection of PLY causes inflammation and tissue damage (80, 81), likely related to PLY-induced corneal epithelial cell lysis (82) (**Figure 1C**).

In the lungs, PLY targets pivotal cell types during acute injury at the early phases of pneumonia and becomes a potent inducer of inflammation, facilitating damage of the respiratory epithelial barrier and host tissue penetration (**Figure 1D**). Purified PLY damages bronchial and alveolar epithelium and slows human ciliary beating *in vitro* (83); it damages alveolar epithelial and endothelial cells impairing barrier function (84, 85); and causes platelet destruction thus inhibiting platelet thrombus formation (86, 87). Anti-PLY polyvalent antibodies were reported to inhibit PLY-mediated platelet annihilation and were proposed to be of pharmacological interest to treat *Sp* community-acquired pneumonia (87). In addition, PLY can compromise effective immune response in the lungs through several mechanisms: it induces the necroptosis of alveolar macrophages (88–90), triggers caspase-dependent cell death of dendritic cells (91, 92), promotes neutrophil transepithelial cell migration and elastase release, thus impairing macrophage phagocytosis, and damages epithelial cells (93–95). The pro-inflammatory role of PLY is discussed in detail in section 4.

If not controlled in the lungs, the bacteria can reach the bloodstream and cause IPD (**Figure 1E**). In the heart, sub-lytic doses of PLY disrupt the PM of cardiomyocytes leading to the influx of Ca^2+^, membrane depolarization and induction of ER stress, which together cause alterations in cardiac rhythm and depression in contractile force. At higher concentrations, PLY causes cardiomyocyte cell death, inhibiting cell contractility and promoting extensive cardiac damage which may lead to microlesion formation and cardiac remodeling likely associated with long-term adverse cardiac events described in IPD patients (34, 96, 97) (**Figure 1F**). *Sp* biofilms, established in the heart of infected mice, release PLY causing rapid macrophage killing and impairing cytokine production (98).

During brain infection, PLY induces neuronal cell death (99, 100) and targets astrocytes, rearranging cytoskeleton and altering astrocyte cell shape, which leads to remodeling of brain tissue, astrocytic retraction, and cortical astroglial reorganization (101–103) (**Figure 1G**). PLY-dependent astrocyte cell death is mediated by connexin 43 (Cx43), a gap junction protein forming hemichannels, that amplifies ATP release and cytosolic Ca^2+^ influx, ultimately leading to astrocyte depletion and blood-brain barrier destabilization (100). PLY also targets other cells in the brain promoting endothelial cell disruption (104), limiting microglia motility (105), and decreasing cilia beating frequency in ependymal cells (106–108), which correlates with loss of cilia and damage of ependymal ultrastructure described in rat meningitis model (109).

## 3 PLY Triggers Multiple Cellular Responses: Irreversible Damage or Repair

**Figure 3:**
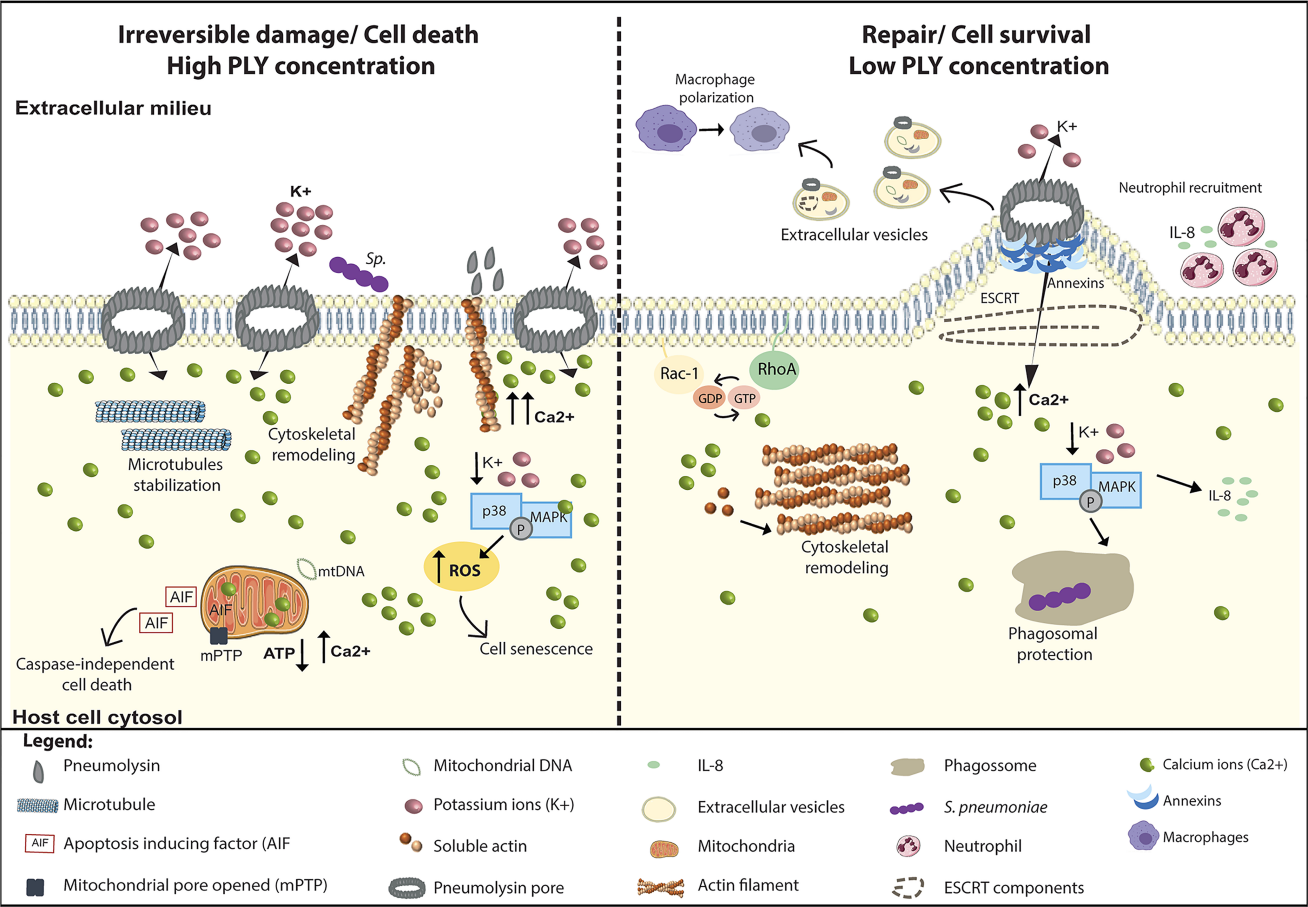
PLY is a trigger for multiple cellular responses. PLY interacts with cells and, depending on PLY concentration and the intracellular Ca^2+^ levels, induces a variety of antipodal cellular responses that can lead to irreversible damage or the induction of cellular repair mechanisms. Left Panel: At lytic amounts, the overwhelming increase in intracellular Ca^2+^ levels induces the surface exposure of actin which facilitate *Sp* adhesion and invasion, increasing cell death. In addition, PLY-mediated microtubule stabilization may perturb axonal transport, likely contributing neuronal damage. Also, in neuronal cells, p38/MAPK activation is detrimental for the host cell as it increases ROS production and induces senescence. High PLY concentrations cause irreversible mitochondrial damage by inducing swelling, loss of mitochondrial membrane potential, and morphologic and metabolic alterations. Concomitantly with Ca ^2+^ overload, mitochondrial permeability increases, the ATP levels decrease, and mitochondrial DNA is released into the cytosol. Following these events, the mitochondrial apoptosis-induced factor (AIF) reaches the cytoplasm and activates caspase-independent cell death. Right Panel: At sub-lytic amounts, the influx of limited amounts of extracellular Ca^2+^ triggers the sequential recruitment of annexins to the damaged sites where they assemble in 3D arrays to clog the PM pore. Increased intracellular Ca^2+^ also induces cytoskeleton remodeling through the activation of small GTPases Rac1 and RhoA and triggers PM rearrangements culminating in PM blebbing and ESCRT-mediated release of microvesicles containing PLY, annexins, actin-binding and Ca^2+^ regulated proteins, ESCRT components and mitochondrial DNA among others. Released microvesicles promote survival by eliminating the pore, transporting danger signals and enhancing immune responses. In response to K^+^ efflux cell survival pathways such p38/MAPK are activated and stimulate the production of pro-inflammatory cytokines such as IL-8, promoting neutrophil recruitment, and enhancing phagosomal integrity, thus limiting the release of toxic bacterial components into the cytosol.

Upon interaction with cells, PLY interferes with a plethora of signal transduction pathways to induce multiple and antipodal cellular responses that define the pathogenesis in a whole organism (**Figure 3**). Such responses are cell-type specific and are dependent on PLY concentration, which correlates with the extent of the PM damage and of the intracellular Ca^2+^ overload. Activation of different cellular responses can tip the balance towards activation of cell death or cell survival pathways, thus promoting tissue damage or repair, respectively. At lytic concentrations, PLY triggers irreversible damage and cell death by causing massive mitochondrial damage, excessive inflammation, and tissue injury. In contrast, at sub-lytic amounts, PLY activates repair pathways triggering PM remodeling, cytoskeleton reorganization, and, by transiently activating MAPK, cell survival.

### 3.1 Mitochondrial Damage

The role of PLY in mitochondrial damage was first described in primary rat neurons infected with *Sp* or incubated with culture supernatants, which both induce the Ca^2+^-dependent mitochondrial release of apoptosis-inducing factor (AIF), a likely manifestation of massive mitochondrial damage (**Figure 3**, left) (110). In addition, PLY was shown to trigger mitochondrial swelling, loss of mitochondrial membrane potential, and impairment in mitochondrial metabolism (111, 112). Electron microscopy analysis of PLY-intoxicated neurons show direct binding of PLY to the mitochondrial membrane (111). In human alveolar epithelial cells, PLY also induces dramatic morphological alterations of the mitochondria, which are accompanied by cytosolic Ca^2+^ overload, reduction in ATP levels, membrane depolarization, increased mitochondria permeability and release of mitochondrial DNA (mtDNA) into the cytosol (**Figure 3**, left) (112). mtDNA can then be released extracellularly through microvesicle shedding and act as a danger signal, thus contributing to inflammation (**Figure 3**, right) (112). In macrophages, cytosolic mtDNA released by PLY-damaged mitochondria is recognized by STING and upregulates the expression and secretion of IFN-β, demonstrated both *in vitro* and *in vivo* in lungs of *Sp* infected mice (113).

### 3.2 Interactions With the Host Cytoskeleton

PLY was reported to bind and promote actin polymerization *in vitro* (114) and was shown to interact with actin filaments underneath the PM of astrocytes (115) or exposed at the surface of damaged neuronal cells (99) (**Figure 3**, left). In fact, PLY D4 domain interacting with cholesterol in the PM of neurons enables β-actin exposure to the outer surface of the cell, facilitating *Sp* adhesion and invasion and increasing cell death (99). In addition, in those cells, PLY induces cytoskeleton instability depolymerizing intracellular actin filaments likely due to increased Ca^2+^ levels (99). How these data relate to the PLY intrinsic ability to polymerize actin *in vitro* needs further investigation, and an understanding of the consequence of PLY-mediated *Sp* interactions with the actin cytoskeleton may require kinetic analysis of the infection in relevant models.

PLY also bundles and stabilizes host cell microtubules during intoxication of neuronal cells and during pneumococcal meningitis in rabbit infection models (**Figure 3**, left) (102). Consistent with increased microtubule stabilization, high levels of acetylated tubulin were found in both the cell culture and animal model (102). PLY-triggered microtubule stabilization is comparable to pharmacological microtubule-stabilizing agents (e.g., taxol) and has been suggested to perturb axonal transport, likely contributing to neuronal damage during pneumococcal meningitis (102). PLY-induced microtubule perturbations are dependent on cholesterol binding and require Src kinase activity but are independent of Ca^2+^ influx and actin remodeling (102).

Several reports, primarily in neuronal cells, suggest that at low concentrations, PLY interaction with host PM causes rapid cytoskeleton remodeling which translates into cell shape changes (**Figure 3**, right). Alterations in the brain structure detected in infected rats were associated with PLY-mediated astrocytes retraction and reshaping of focal adhesions, which are underlined by cytoskeleton reorganization (101, 103). In primary mouse astrocytes and neuronal cells, sub-lytic concentrations of PLY binding to cholesterol promotes the formation of actin stress fibers, filopodia, and lamellipodia through the activation of RhoA and Rac1 GTPases (**Figure 3**, right) (114, 115).

### 3.3 Activation of Cell Survival Pathways

PLY binding to the PM triggers activation of cell survival pathways such as the mitogen-activated protein kinase p38 (p38/MAPK) (116, 117), which is a conserved response to sub-lytic doses of PFTs dependent on K^+^ efflux (118), that can stimulate cell survival pathways (117). In epithelial cells, activation of p38/MAPK by sub-lytic concentrations of PLY (119, 120) triggers the production of pro-inflammatory cytokines (e.g., IL-8) to attract neutrophils, thus promoting an effective immune response early in infection when bacterial numbers are low (**Figure 3**, right) (119). In macrophages, PLY-induced p38/MAPK activation and cytokine production take place at the PM upon pore formation (119, 120) and has been suggested to promote *Sp* clearance (121). Later in infection, following *Sp* uptake by macrophages, PLY induces the trafficking of pneumococcal cell wall components to the host cell cytosol, presumably through phagosomal damage (122). If p38/MAPK activation is blocked, leakage of *Sp* cell wall components to the host cell cytosol is exacerbated and results in macrophage cell death. Thus, PLY-induced p38/MAPK activation protects phagosomal integrity and limits the release of bacterial components, likely modulating the recognition of pathogen-associated molecular patterns by the host surveillance systems (**Figure 3**, right) (122, 123).

The timing of the MAPK response to PFTs can influence tissue injury. Although MAPK activation can promote cell survival responses upon intoxication by PFTs, its subsequent modulation by protein phosphatases PP1 and PP2A, observed in epithelial cells (120), can prevent excessive inflammatory responses that could lead to irreversible tissue damage. Conversely, the transient nature of p38/MAPK activation might not be sufficient to block cell death triggered by the recognition of *Sp* components in the macrophage cytosol (122).

Finally, in some cell types, activation of p38/MAPK is detrimental. In SH-SY5Y neuronal cells, p38/MAPK is associated with increased neuronal cell death and neurotoxic effects (124), and in microglial, PLY-mediated MAPK activation increases ROS production and promotes senescence (**Figure 3**, left) (125).

### 3.4 Activation of Plasma Membrane Repair Mechanisms

Supernatants from stationary phase cultures of *Sp* produce sufficient amounts of PLY to permeabilize cells (126); however, during infection, the majority of these perforated cells are able to recover from damage and survive. PLY pore assembly renders host cell PM permeable to ions and small molecules (116, 127, 128). An increase of Ca^2+^ concentrations to above 20 μM impairs host-cell signaling and engages cell death pathways (116, 129). However, an initial increase in intracellular Ca^2+^ levels act as a danger signal and activates PM repair mechanisms to prevent cell lysis (119, 124, 130–132). Thus, at otherwise sub-lytic PLY concentrations, reduced extracellular Ca^2+^ enhances PLY toxicity and result in lysis (133, 134). In the presence of extracellular Ca^2+^, PLY-induced Ca^2+^ influx triggers the recruitment of annexins, cytoplasmic Ca^2+^ responsive proteins that bind to negatively charged phospholipids at the sites of PM injury (135). Annexin A2, which displays the highest Ca^2+^ sensitivity, is the first to translocate to the site of damage, followed by annexin A6 and A1 (**Figure 3**, right). Annexin translocation fosters the formation of plasmalemmal nanotubes at the pore site that culminates in the release of microvesicles enriched in PLY, annexins, actin-binding and Ca^2+^ regulated proteins, and ESCRT components (**Figure 3**, right) (126, 134), thus shedding the PLY pore and modulating the rise in intracellular Ca^2+^ (129). This repair process has potential immunological implications for *Sp* infection, as PLY-containing microvesicles induce macrophage polarization that enhances the immune response towards molecular patterns of Gram-positive bacteria (136). By contrast, in alveolar epithelial cells, human lung explants and infected mice, PLY mediates the release of microvesicles containing mitochondrial cargo that, when uptake by neutrophils suppress their ability to release ROS and thus impair efficient immune response against *Sp* (137). The differential PLY sensitivity of immune cells is thought to be due to differential efficiency in PM repair (138). Myeloid cells, the first-line defenders, show enhanced shedding of PLY-containing microvesicles and increased resistance to PLY. In contrast, lymphoid cells are enriched in cholesterol-containing lipid rafts, which facilitate PLY binding and pore formation, and are impaired in microvesicles formation and PM resealing activity, leading to high PLY susceptibility (138).

## 4 PLY Interactions With the Immune System: Pro- or Anti- Inflammatory Responses

**Figure 4:**
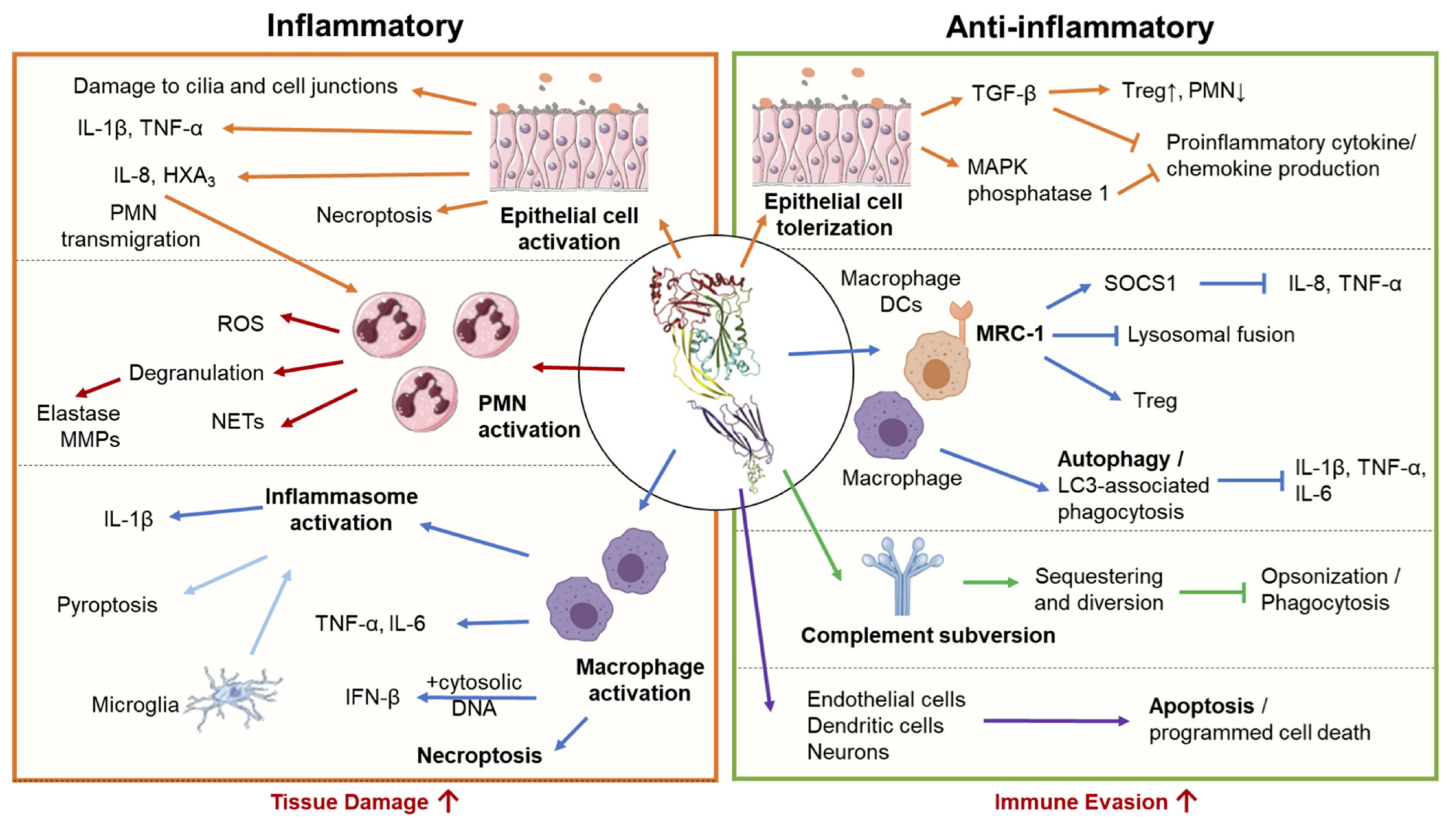
Pro-inflammatory and anti-inflammatory host responses to PLY. *S. pneumoniae* triggers both pro-inflammatory and anti-inflammatory responses depending on interacting host cell type and infection context. Left Panel: In excess, pro-inflammatory actions of PLY enhance tissue damage and promote bacterial spread. In epithelial cells, PLY induces production of pro-inflammatory cytokines and chemokines, promoting neutrophil transmigration and compromising epithelial barrier function. PLY-stimulated neutrophils engage in a wide range of effector functions, including degranulation, reactive oxygen species (ROS) production, and neutrophil extracellular trap (NET) release, many of which propagate inflammatory tissue damage and are associated with severe pathology in the lung. PLY can activate macrophages independently or in conjunction with other co-stimulants to cause the release of pro-inflammatory cytokines and chemokines. Macrophages can also be activated by PLY-dependent inflammasome activation, which in bone marrow-derived macrophages leads to IL-1β -mediated inflammation, and in microglia, pyroptotic cell death. PLY is also a potent inducer of macrophage necroptosis, often leading to acute tissue injury. Right Panel: Anti-inflammatory activities of PLY downregulate immune responses and may aid bacterial evasion. During pneumococcal colonization and early stages of lung infection, PLY suppresses inflammatory cytokine production by airway epithelial cells and enhances recruitment ofT regulatory cells, promoting unchecked bacterial colonization. Internalization of PLY by alveolar macrophages and dendritic cells via the mannose receptor MRC-1, or, in bone marrow derived macrophages, PLY triggered LC3- associated phagocytosis, suppress the production of inflammatory cytokines. To avoid complement­ mediated detection and opsonization, PLY acts as a decoy molecule to sequester complement proteins. Finally, PLY triggers apoptosis in a wide range of cell types, including endothelial cells, neurons, and dendritic cells, allowing for non-inflammatory removal of these cell types.

PLY can either exacerbate or mitigate damage during infection depending on the state of the immune system as well as the infection site, dose, timing, and interacting host cell type (26, 139). It can trigger inflammation-mediated tissue damage and promote bacterial dissemination (18, 140, 141). However, PLY can elicit host-protective responses in the innate and adaptive branches of the immune system, including activation and stimulation of cytokine production by macrophages, neutrophils, endothelial, epithelial, and dendritic cells (22, 142–144). The multifaceted nature of PLY-induced immune responses may account for some of the disparate outcomes of infection by PLY-deficient strains in different infection models (**Figure 4** and **Table 1**). This also reflects the dual relationship of *Sp* with host cells, sometimes invasive and inducing severe disease, and other times remaining localized as an asymptomatic colonizer (as discussed in sections 1 and 2.4.).

**Table 1:** PLY-triggered pro- and anti-inflammatory immune modulations.

Nature of PLY-triggered immune response	Immune processes activated	Target host cell or factor type	Specific actions	Effect on infection outcome
**Pro-inflammatory**	**Epithelial cell activation**	Epithelial cells	Disruption to cilia organization and movement Disruption to cell junction complexes	Compromised airway epithelial barrier function
			Release of pro-inflammatory cytokines IL-1β, TNF-α Release of chemokines IL-8, and HXA_3_	Increased tissue damage
	**Neutrophil activation**	Neutrophils	Release of chemokines IL-8, f-MLP, PLA2s, PGE2, LTB_4_ Increased neutrophil transmigration Prolonged neutrophil accumulation	Increased tissue damage and systemic spread
			Enhanced pro-inflammatory secretory profile: ROS, serine proteases, MMPs Increased NETosis	Disrupted ECM, surfactants and cell junction proteins increasing lung permeability Increased systemic spread
	**Inflammasome activation**	Macrophage and dendritic cells	NLRP3 or AIM2 triggered inflammasome-dependent cytokine production: IL-1α, IL-1β, and IL-18	Enhanced bacterial clearance
		Microglia	Pyroptotic death	Increased neurotoxicity
	**Macrophage activation**	Macrophage	Enhanced pro-inflammatory secretory profile: IL-1β, IL-6, TNF-α, IFN-β, IL-23, GM-CSF, MIF, NO, and pro-inflammatory exosomes	Enhanced bacterial clearance and immune cell recruitment
	**Necroptosis**	Tissue resident macrophages and cardiomyocytes	Alveolar macrophage depletion Cardiac macrophage depletion and cardiomyocyte death	Increased tissue injury
		Epithelial cells	Epithelial cell death	Anti-*Sp.* antibody production
**Anti-inflammatory**	**Tolerization to colonization**	Epithelial cell	TGF-β secretion Suppressed TNF-α production	Increased Treg activity and reduced neutrophil infiltration Increased nasopharyngeal colonization
			Downregulation of *Sp.-*detecting receptors: CD21, PAFR, OLR1	Nasopharyngeal colonization evading immune detection
	**Anti-inflammatory polarization through MRC-1**	Alveolar macrophage and dendritic cells	SOCS1-mediated suppression to TNF-α Decreased lysosomal fusion	Decreased cytokine production Increased bacterial burden
	**Autophagy**	Epithelial cells, fibroblasts, and macrophages	Canonical autophagy and/or LC3-associated phagocytosis	Decreased cytokine production Increased intracellular bacterial clearance
		Microglia	Delay of caspase-1 activation and pyroptotic death	Decreased neurotoxicity
	**Complement subversion**	Complement proteins	Sequestration of complement components by binding to immunoglobulins (classical pathway) and L-ficolin (MBL pathway)	Decreased serum opsonic activity
	**Apoptosis**	Neurons, endothelial cells, cochlear hair cells, and dendritic cells	Mitochondrial damage-associated release of AIF	Decreased inflammatory cytokine production
		Epithelial cells	DNA damage-induced cell cycle arrest	Depletion of responding immune cells
		Macrophages and dendritic cells	Phagolysosome membrane disruption-induced caspase activation

### 4.1 Pro-Inflammatory Properties

#### 4.1.1 Epithelial Cell Activation

Epithelial cells are a first line of defense against *Sp*, in part by functioning as a physical barrier against invasion and, in the lower respiratory tract, maintaining the mucociliary elevator that removes microbes from the lung. PLY both disrupts epithelial cell junctions, compromising this barrier, and interfering with the number and organization of cilia in human and mouse airway epithelium-derived air-liquid interface organoid cultures (145, 146), crippling the mucociliary elevator. In addition, as the sentinels for infection, the mucosal epithelium produces a range of antimicrobial peptides and pro-inflammatory signaling molecules to eliminate invading pathogens and alert immune cells to response to invasion. PLY induces β-defensin 2 secretion in A549 human lung epithelial cells and in human middle-ear cells (147). PLY, is also a potent activator of cytokine and chemoattractant secretion through pore forming insult to airway epithelial cells (94, 119, 148) (**Figure 4**, left). PLY induces epithelial cell release of IL-1β, TNF-α (148), and the neutrophil chemoattractants IL-8 (119) and hepoxilin A_3_ (HXA_3_) (94, 149, 150). Accumulation of inflammatory cytokines and chemokines facilitate the recruitment of leukocytes responding to and clearing *Sp*. Nevertheless, in both a lung epithelial tissue culture and a mouse pulmonary infection model, HXA_3_-mediated basolateral-to-apical neutrophil transmigration exacerbates damage to epithelial barrier and promotes lethal *Sp* systemic spread (151, 152).

#### 4.1.2 Neutrophil Activation

As described above, neutrophil infiltration and the associated epithelial damage promote bacterial dissemination into the bloodstream, resulting in lethal systemic disease (151). While neutrophil recruitment to the lungs during early stages of infection aids *Sp* clearance, overtime, reduction in adenosine production by lowered CD73 expression correlated with loss of neutrophil antimicrobial efficacy (153). Extracellular ATP is shown to neutralize PLY-mediated neutrophil activation and may explain this shift in neutrophil response profile (154). Excessive neutrophil activities can be harmful to the host (140). Indeed, the prolonged accumulation of PMNs in a murine model of pneumococcal pneumonia results in increased burden and high tissue damage (153). Upon extravasation and encountering invading bacteria, exposure of PMNs to PLY can trigger the release of additional neutrophil-recruiting signals, including Ca^2+^-dependent increases in IL-8, f-MLP, phospholipase A, A2, prostaglandin E_2_, and leukotriene B_4_ (155, 156), reinforcing neutrophil-direction inflammation. Neutrophils at the site of infection can be further activated by *Sp* and PLY to produce reactive oxygen species (ROS) (93, 142, 157), degranulate, and form neutrophil extracellular traps (NETs) (**Figure 4**, left).

ROS have multiple immune-modulatory functions during bacterial infection: in addition to direct antimicrobial activity and signaling to modulate immune cell function, ROS can also cause damage to host cells and tissues (158). Interestingly, pretreating neutrophils with PLY promotes sensitivity to f-MLP (157), which conditions neutrophils to release higher and more sustained levels of ROS, and become more prone to degranulation (**Figure 4**, left) (142).

During degranulation, neutrophils secrete proteolytic enzymes capable of propagating tissue damage. These include serine proteases neutrophil elastase (NE), cathepsin G and proteinase-3, and neutrophil metalloproteinases (MMPs). PLY can trigger NE release either by degranulation of primary granules or by direct cell lysis (93, 142). Although NE degrades pneumococcal cell wall-localized aminopeptidase N and can facilitate opsonophagocytic killing in the presence of an antiphagocytic capsule (93, 159–161), excessive NE activity is detrimental to the host (162). NE has potent catalytic activity against a range of extracellular matrix proteins, including elastin, proteoglycan, fibronectin, and several collagen types (163), surfactant proteins (162), and alveolar epithelial cell junction protein such as E-cadherin (164). In addition, NE stimulates the lung epithelium to release proinflammatory cytokines and induces epithelial apoptosis (165–167). Severe pneumonia in human patients and experimental animals is associated with increased NE levels in lung bronchoalveolar lavage fluid (168, 169) and plasma (170). NE may thus be a critical inducer of epithelial permeability during pneumococcal pneumonia, compromising lung epithelial barrier and promoting bacterial dissemination (171). Cathepsin G and proteinase-3, which like NE are stored in azurophilc granules, also contribute to infection-related lung injury, albeit to a much lesser degree than NE (172). Metalloproteinases (MMPs) are neutrophil granule components that are also released following the increase in cytosolic Ca^2+^ levels resulting from PLY-mediated pore formation and can be augmented by concurrent stimulation by f-MLP (173). MMPs, especially MMP-8 and -9, have been correlated with tissue injury in pneumococcal pneumonia and meningitis (174–176). Thus, the PLY-mediated release of neutrophil granule components contributes to inflammation and pathogenesis, especially during severe forms of *Sp* disease.

Finally, pneumococcal capsule and PLY work in synergy to promote neutrophil extracellular trap (NET) production (162), a vital neutrophil effector function for trapping and killing extracellular microbes (**Figure 4**, left). While NETs exhibit significant antibacterial activity against *Sp* (177), their efficacy is counteracted by *Sp*-secreted endonucleases EndA and TatD that digest NETs and facilitate escape (162). NETs are intercalated with NE and other proteases that can damage host cells just as they can a pathogen, and excessive NETosis against *Sp* is implicated in promoting lung injury (178), sepsis (179), and increasing mortality (180). PLY is thus a potent activator of multiple neutrophil effector functions, most of which can propagate inflammatory tissue damage in the lung and are associated with severe pathology.

#### 4.1.3 Inflammasome Activation

PLY induces inflammasome-dependent IL-1β secretion and initiates the pro-inflammatory cascade associated with *Sp* infection (181–183). PLY-mediated K^+^ efflux, which is sufficient to trigger NLRP3 activation (184), results in caspase-1 cleavage and the consequent production and secretion of IL-1β (181, 185). An alternative inflammasome activation pathway aided by PLY is the AIM2 inflammasome pathway. AIM2 senses double-stranded DNA from lysed bacteria, which can localize to host cell cytosol after PLY-mediated phagosomal membrane disruption (186, 187). PLY-deficient *Sp* induces less inflammasome-dependent cytokines production (including IL-1α, IL-1β, and IL-18), which influence downstream cytokine and chemokine responses to *Sp*. Indeed, inflammasome-dependent cytokines promote secretion of IL-17A, IFN-γ, and neutrophil chemoattractants, each of which aids bacterial clearance (123, 182, 188). Inflammasome activation is host protective in mouse and human macrophages *ex vivo* and in mouse pneumococcal infection models (182, 188, 189). *Sp* strains impaired in inflammasome activation, including some serotype 1, serotype 8, serotype 7F, and strains expressing the nonhemolytic allele 5 of PLY, tend to cause more invasive disease (189–191) and chronic infection (17). In contrast, in pneumococcal meningitis models, PLY-dependent inflammasome activation increasing neurotoxicity and pathology (192), possibly because PLY-triggered inflammasome activation results in rapid pyroptotic death in microglial cells (193). Thus, while inflammasome activation is in general a protective response against *Sp* infection, tissue types such as the brain can be susctiple to pathologies due to pyroptotic death (**Figure 4**, left).

#### 4.1.4 Macrophage Activation

PLY activates macrophages to release of IL-1β, IL-6, TNF-α, IFN-β, IL-23, granulocyte-macrophage colony-stimulating factor (GM-CSF), macrophage migration inhibitory factor (MIF), and nitric oxide (NO) (194–197). Many of these pro-inflammaroty and chemotactic responses rely on p38 or inflammasome activation. Some PLY-induced macrophages responses require additional *Sp* factors as co-stimulants (198). For instance, phagosomal membrane disruption by PLY allows for pneumococcal DNA to enter the cell cytoplasm, triggerring cytosolic DNA sensors for IFN-β production (113). PLY can also modulate macrophage activation when sequestered into vesicles shed during membrane repair; interaction with PLY-containing vesicles increases macrophage IL-1β, TNFα, CCL5, CCL8, and CCL1 (136).

In the monocyte-derived THP-1 cell line, PLY is responsible for the majority of gene modulations upon exposure to *Sp* (194). Upregulated genes include those encoding proinflammatory molecules such as IL-8 and monocyte chemotactic protein 3 (MCP-3), and cell surface receptors that impact inflammation, such as macrophage inflammatory protein 1β (MIP-1β), IL-2 receptor β (IL-2Rβ), IL-15 receptor α (IL-15Rα), and interferon receptor 2, promoting pro-inflammatory cytokine propagation (**Figure 4**, left).

#### 4.1.5 Necroptosis of Multiple Cell Types

PLY induces necroptosis, an inflammatory pathway of programmed cell death, in the lungs (90) and heart (199). In the lungs, PLY-mediated necroptosis of alveolar macrophages (90) and epithelial cells (90) is triggered in response to loss of ion homeostasis, ATP depletion, and ROS generation (90), and depends on the phosphorylation of MLKL (200), a master effector of necroptosis. The rapid depletion of alveolar macrophages by necroptosis may greatly contribute to extensive lung damage (**Figure 4**, left). In the heart, PLY-mediated necroptosis occurs in macrophages that infiltrate the infected myocardium and in cardiomyocytes, thereby suppressing the anti-*Sp* immune response at that site (199) and contributing to cardiac injury (201). The use of pharmacological inhibitors of necroptosis reduces acute injury in the lungs and heart during *Sp* infection, providing a novel therapeutic target to overcome infection (201). Interestingly, PLY-induced necroptosis was also reported in nasopharyngeal epithelial cells during *Sp* asymptomatic colonization and was associated with the increased production of anti-pneumococcal antibodies (202), suggesting a role in the development of protective immunity against *Sp* and highlighting the infection contex dependent nature of PLY-host interactions.

### 4.2 Anti-Inflammatory Properties

#### 4.2.1 Epithelial Cell Tolerization

As a prerequisite to becoming a deadly pathogen, *Sp* asymptomatically colonizes the human nasopharynx. This immune-silent colonization and long-term *Sp* carriage are thought to be sustained by anti-inflammatory responses, which prevent the disruption of the epithelial barrier and the infiltration of neutrophils (203). After nasopharyngeal inoculation of 10^5^ *Sp* in mice, a dose that results in asymptomatic carriage, the production of PLY is associated with higher levels of the immunosuppressive cytokine TGF-β1 and immunomodulatory T regulatory cells (Tregs) and lower levels of neutrophil recruitment (**Figure 4**, right). *Sp* infection of human nasopharyngeal epithelial cells and fibroblasts resulted in PLY-dependent secretion of TGF-β1. Treatment of these cells with purified PLY likewise results in TGF-β1 secretion (203). In contrast, compared to the 10^5^ dose, nasopharyngeal inoculation of 10^7^ *Sp*, which leads to bacterial clearance, results in the production of lower levels of TGF-β1 and Tregs, and higher levels of IFN-γ and neutrophil infiltration (203). These findings indicate that PLY is capable of fostering nasopharyngeal carriage by limiting proinflammatory responses.

Interestingly, compared to other bacterial respiratory pathogens, such as *Haemophilus influenzae*, *Sp* induced cytokine production by epithelial cells *in vitro* appears delayed (148), consistent with the low numbers of infiltrated neutrophils detected in early stages of lobular pneumonia in human patients, despite bacterial load (204). At these early infection stages, PLY promotes the expression of MAPK phosphatase 1, which dephosphorylates p38 to suppress TNF-α production (205) (**Figure 4**, right). Later during infection, secretion of pro-inflammatory cytokines by epithelial cells increase (148). This delay in the initiation of epithelial cell-mediated inflammation may delay immune cell infiltration and provide *Sp* with a window of unchecked growth to establish infection. In addition, PLY downregulated many binding receptors that aid *Sp* detection by macrophages, including complement component receptor 2/CD21, platelet-activating factor acetylhydrolase, and oxidized low-density lipoprotein receptor 1 (OLR1). Thus, on top of directly targeting epithelial cells for tolerization, PLY evades surveilling immune cells by downregulating critical receptors for *Sp* recognition.

#### 4.2.2 MRC-1 as a Mediator of Anti-Inflammatory Response

As mentioned, PLY was recently described to directly interact with MRC-1, which is expressed by DCs and alveolar macrophages, two cell types that represent a first line of defense mounted against *Sp* in the lungs (61). The PLY-MRC1 interaction impairs inflammatory response, limiting inflammatory cytokine secretion through the cytokine suppressor SOCS1, as well as neutrophil infiltration (**Figure 4**, right) (61). Consistent with its role as a phagocytic receptor, MRC-1 expressed on DCs binds to sub-lytic concentrations of PLY and promotes internalization of PLY-producing *Sp*. In mouse alveolar macrophages, internalized PLY-producing *Sp* colocalizes with MRC-1, whereas PLY-deficient *Sp* colocalizes with lysosomes (**Figure 4**, right). Upon pulmonary challenge of mice, PLY production is associated with lower levels of TNF-α and greater numbers of bacteria in lavage fluid. Similarly, genetic ablation or antibody inhibition of MRC-1 results in higher levels of TNF-α and lower bacterial load (61).

These findings opened new therapeutic strategies to fight pneumococcus infection. Indeed, molecular docking approaches identified MRC-1-derived peptides that neutralize PLY-MRC-1 interaction that impair *Sp* internalization and promote pathogen killing by autophagy (206). Furthermore, MRC-1-derived peptides were shown to inhibit PLY-driven cell lysis, inflammation, and lung epithelium damage by limiting PLY interaction with cells and decreasing IL-8 and TNF-α secretion (206). In zebrafish and mouse models of pneumococcal infection, MRC-1-derived peptides reduce disease development, promote host survival, and decrease bacterial burden (206).

#### 4.2.3 Triggering of Autophagic Processes

Autophagy is a natural self-degradative mechanism that is activated in epithelial and immune cells to degrade cytoplasmic content. Autophagy also plays major roles in pathogen elimination controlling both inflammation and adaptive immune response (207). *Sp* activates autophagy, which functions as a host protective mechanism by promoting *Sp* clearance (208). As anticipated by a membrane-damaging agent, PLY was shown to initiate autophagy in a variety of cells such as human alveolar epithelial cells (208), murine microglia (193), and osteoblast cells (209). ROS generation triggered by PLY leads to the inhibition of the PI3K/AKT/mTOR pathway and the consequent activation of canonical autophagy (193, 208), which degrades intracellular *Sp* and limits infection in host cells (208). In murine microglia, autophagy activated by *Sp* transiently blocks caspase-1 activation and IL-1β secretion, delaying pyroptosis, the caspase-1-dependent inflammatory cell death pathway (193). In these cells, *Sp* infection increases the expression of autophagy-related genes in the early phase of infection as a host protective mechanism before pyroptosis and concomitant extensive tissue damage can occur (**Figure 4**, right). However, at later stages of infection, sustained PLY-mediated high-level ROS generation activates caspase-1 and microglia pyroptosis (193). In osteoblasts, PLY-mediated activation of autophagy impairs differentiation by regulating the expression of differentiation-related genes, which require mTOR signaling (209), a finding with potential relevance to *Sp* osteomyelitis. Finally, PLY induces selective autophagy promoting the delivery of *Sp* entrapped in autophagosomes to lysosomes for further degradation (210). In fibroblasts, PLY triggers (LC3)-associated phagocytosis (LAP), followed by canonical autophagy activation, suggesting a hierarchical autophagy activation process leading to *Sp* clearance (211). In fibroblasts, the activation of canonical autophagy is required for *Sp* degradation, but in bone-marrow-derived macrophages (BMDMs), PLY-induced LAP is sufficient for bacterial clearance, allowing degradation to occur much more rapidly (212). In aged BMDMs, LAP is compromised, and cells display reduced *Sp* killing capacity and increased expression of proinflammatory cytokines (**Figure 4**, right) (212).

#### 4.2.4 Complement Subversion

Complement activation has a crucial role in host protection against pathogens, but *Sp* utilizes PLY-mediated complement activation to sequester complement components away from *Sp* surface, thus protecting *Sp* from host defenses and facilitating bacterial spread and survival. Purified PLY activates the human complement cascade *via* the classical pathway independently of its lytic activity (62, 213). Homology domains shared with CRP, mediate the PLY binding to the Fc portion of immunoglobulins, which in turn recruit and activate C1q, and are required for PLY-triggered complement activation (62, 63). While in serum from C1q KO mice PLY fails to trigger complement activation, in human C1q-depleted serum, PLY still activates the complement and C3b deposition is observed (214), suggesting that in humans, PLY may stimulate complement through C1q-independent pathways that directly target C3. PLY was further shown to trigger lectin pathway by binding to L-ficolin with high affinity (214). However, PLY-triggered complement activation decreased the serum opsonic activity for *Sp* both *in vitro* and *in vivo* in mouse models, reducing bacterial uptake by neutrophils and impairing the recruitment of T cells to the sites of infection in the lung (62, 64, 213).

#### 4.2.5 Induction of Apoptosis

Pathogen-induced apoptosis, a non-inflammatory programmed cell death pathway, plays an important role in tissue damage caused by infectious diseases and constitutes an important mechanism of protection from invasive disease. PLY cytolytic activity can prompt cells to engage in apoptosis by different mechanisms depending on PLY concentration and the cell type involved (**Figure 4**, right). One of the first studies connecting PLY to apoptosis showed that in neurons, PLY pore-forming activity triggers Ca^2+^ influx and mitochondrial damage which results in the release of pro-apoptotic factor (AIF) and induces apoptosis in a caspase-independent manner (110, 111). The same mechanism was described in brain microvascular endothelial cells, inner cochlear hair cells, and DCs (77, 91, 215). In addition, intracellular PLY induces caspase-dependent apoptosis in *Sp*-infected human dendritic cells, whereby blocking their maturation and the production of inflammatory cytokines, thus inhibiting dendritic cell-mediated inflammatory responses (92). Caspase-dependent apoptosis, triggered by PLY-induced phagolysosomal membrane permeabilization, also occurs in human monocyte‐derived macrophages (216, 217). In endothelial cells, PLY-triggered caspase activation and apoptosis depend on the activation of p38/MAPK and suppression of extracellular signaling regulation kinase (ERK)1/2 (218). Further supporting PLY’s ability to trigger apoptosis, in human epithelial lung alveolar cells PLY causes double-stranded DNA breaks, which led to cell cycle arrest followed by non-homologous end joining DNA repair or, if DNA damage persists, by engagement in apoptosis (219). Clinical isolates deficient for PLY are unable to induce DCs apoptosis and trigger a strong proinflammatory response leading to excessive lung inflammation (92). Thus, the deleterious effect of PLY on immune cells, blocking their maturation, inhibiting the secretion of inflammatory cytokines, and inducing apoptosis, promotes bacterial evasion from immune detection and allows for immune-silent colonization.

## 5 Future Perspectives

A better understanding of the complex “Yin” and “Yang” properties of PLY will facilitate the translation of critical *Sp* pathogenesis mechanisms into clinical intervention strategies. PLY stands as an attractive therapeutic target to complement classical antibiotic therapy, which, besides fostering the development of resistance, can trigger bacterial lysis and consequent release of PLY that alone can have deleterious effects on cells and the immune system (25). Several repurposed drugs and PLY-neutralizing compounds have been investigated. Statins, which inhibit cholesterol production, administered prior to infection both *in vitro* or *in vivo* confer significant resistance to PLY cytotoxicity by impairing binding (220, 221). Also, some natural compounds were shown to target the oligomerization process by binding to specific D3 and 4 residues, resulting in reduced cytolytic activity (222–226). The use of artificial liposomes was also suggested as a way to sequester PLY. The administration of liposomes prior to pneumococcal infection reduced septicemia and invasive disease in a mouse model (171). Recent efforts have been made to find novel therapeutic tools to inhibit PLY release, and recently, a study demonstrated that clarithromycin downregulates ply transcription *in vitro* and *in vivo* and consequently reduces PLY production by bacteria (227), proposing a new strategy to treat pneumococcal disease. The clinical potential of current PLY-neutralizing therapeutic strategies and their limitations have been extensively reviewed elsewhere (22, 25).

Since PLY is also a potent trigger for anti-*Sp* immune responses, harnessing the immunogenic potentials of PLY has long been of therapeutic interest. PLY-immunized mice displayed significantly increased survival upon infection, and thus suggested that PLY should be considered for inclusion in a human vaccine (228). Further studies using different animal models aimed to obtain an active immunization using a genetic toxoid derivative of PLY, alone or in combination with other proteins (229–234). In fact, PLY toxoid demonstrated potential effectiveness as an immunogenic component and in controlling bacteremia and bacterial colonization (229–235). Phase I clinical trials concerning the use of PLY toxoid vaccine formulations were performed in adults and children, and results demonstrated that it is well-tolerated and immunogenic when administered as individual protein vaccines or combined with capsule polysaccharide conjugates (230, 236, 237). Experimental evolution studies revealed the emergence of variants that produce low levels of PLY showing decreased virulence but increased persistence (238). Such lineages have been proposed to serve as starting point to the development of live-attenuated pneumococcal vaccines.

To use PLY-targeting therapies and treatment tailored to the stage and severity of pneumococcal infection, requires a better elucidation of the role of PLY in pathogenesis. As highlighted in this review, PLY induces multiple contradicting responses. The parameters that dictate the exact mechanisms triggered in the host cells during pore-formation are still elusive. It is known that PLY is present in circulation during early pneumococcus infection and sub-lytic doses induced toxicity and modulated host immune response; furthermore, host cells can trigger PM repair mechanisms to recover from damage and get rid of the toxin (116, 127, 130, 239). However, it is still not clear which molecular mechanisms and host proteins are involved during the process of cell survival and the importance of these events in the context of infection. Cells can expel the toxin through vesicle shedding, but whether they can function as a danger signal to neighboring cells or as a modulator of the immune response remains unclear (126, 129, 136, 138). PLY can also have several effects in intracellular signaling and modulate host cytoskeleton but remains uncertain how these events can help cells to survive and repair the damage (101, 103, 114, 115, 240). Furthermore, the use of complex models like 3D cell culture or *in vivo* models should now be considered to clarify the crosstalk between different host cell types and their microenvironment. Insights into the mechanistic control for cellular response to PLY pores may also help resolve the apparent contradictory inflammatory and anti-inflammatory implications under different infection contexts. The discovery of new mechanisms of host PM repair and survival may bring new insights to the development of therapies against the injurious actions of PLY during pneumococcus infection.

## Author Contributions

All authors listed have made a substantial, direct and intellectual contribution to the work, and approved it for publication.

## Funding

SS research receives funds from FEDER—Fundo Europeu de Desenvolvimento Regional funds through the COMPETE 2020—Operational Programme for Competitiveness and Internationalization (POCI), Portugal 2020, and by Portuguese funds through FCT—Fundação para a Ciência e a Tecnologia/Ministério da Ciência, Tecnologia e Ensino Superior in the framework of the project POCI-01-0145-FEDER-030863 (PTDC/BIA-CEL/30863/2017). Research in JL’s laboratory is supported by NIH R21 AG071268-01. JMP is the recipient of an FCT fellowship (SFRH/BD/143940/2019). SS received support from FCT in the framework of CEEC-Institutional 2017 program.

## Conflict of Interest

The authors declare that the research was conducted in the absence of any commercial or financial relationships that could be construed as a potential conflict of interest.

## Publisher’s Note

All claims expressed in this article are solely those of the authors and do not necessarily represent those of their affiliated organizations, or those of the publisher, the editors and the reviewers. Any product that may be evaluated in this article, or claim that may be made by its manufacturer, is not guaranteed or endorsed by the publisher.
